# The Impact of COVID-19 on Medical Research: Perceived by Faculty of Medical and Health Sciences Colleges in the University of Sharjah

**DOI:** 10.7759/cureus.86850

**Published:** 2025-06-27

**Authors:** Hoor A Aldarraji, Albara Alshalkhaty, Othman M Asaad, Gana Ali Tahmaz, Raghad A Al saleh, Amal Hussein

**Affiliations:** 1 College of Medicine, University of Sharjah, Sharjah, ARE; 2 Family and Community Medicine, University of Sharjah, Sharjah, ARE

**Keywords:** covid-19, epidemiology, public health, research, uae

## Abstract

Background

The role of medical research has increased in the wake of the COVID-19 pandemic, which imposed several limitations, including a stay-at-home policy, lockdown, and travel restrictions. These constraints strained the research system, making participant recruitment and data gathering difficult. The study highlights significant disruptions in research activities among faculty at the University of Sharjah during the COVID-19 pandemic, underscoring the need for improved institutional strategies to support research continuity during future health crises.

Methods

A cross-sectional study was conducted among 101 participants who completed a self-administered questionnaire comprising three main sections: demographics, research activities, and the impact on research conduction. Data was collected via email and analyzed using SPSS version 22.0. A Chi-square test with a 5% significance level was employed.

Results

Out of 101 participants, 61.39% were men, and around 45% were aged 42-50 years. Most were established researchers from the College of Medicine. Of these, 45% conducted lab work research. COVID-19 significantly impacted research stages, particularly subject recruitment. Researchers in cross-sectional and case-control studies felt less threatened about losing their research. Senior researchers were 5.68 times more likely to face access issues to facilities, archives, or medical centers compared to established researchers. Gender, college, researcher level, and study design type had no significant impact on the number of research projects.

Conclusion

The study highlights significant disruptions in research activities among faculty at the University of Sharjah during the COVID-19 pandemic, underscoring the need for improved institutional strategies to support research continuity during future health crises.

## Introduction

Medical research plays a critical role in advancing healthcare across the globe. Its importance has been especially evident during the COVID-19 pandemic, where rapid scientific investigation has guided physicians in understanding and managing the crisis. The pandemic has highlighted the critical role of research, underscoring the need for timely, evidence-based responses to global health threats. In this context, the World Health Organization has urged researchers worldwide to examine the impact of COVID-19 and pursue health-related studies that can inform public health strategies and improve outcomes.

This global momentum also extends to the United Arab Emirates, where research efforts continue to grow in response to the evolving healthcare landscape. The United Arab Emirates (UAE) has strongly committed to advancing medical research through strategic initiatives and supportive government policies. The Science, Technology, and Innovation Policy focuses on key healthcare areas, including cardiovascular disorders and cancer sciences, aiming to position the UAE as a leader in medical innovation [1]. Additionally, the Research and Development Governance Policy, launched in 2021, seeks to enhance the efficiency and effectiveness of research activities across the nation. These efforts have contributed to an 86.5% increase in medical research activities between 2014 and 2018, underscoring the UAE’s emergence as a significant hub for medical research [2].

The COVID-19 pandemic has profoundly disrupted clinical research worldwide. A study analyzing data from March to April 2020 identified over 1,000 clinical trials suspended, with at least 905 attributing the suspension directly to the pandemic [3]. This trend was observed globally; for instance, in April 2020, regions such as North America and Europe experienced a 70.2% and 72.9% decrease in newly initiated non-COVID-19 clinical trials compared to the same period in the previous year [4]. Additionally, patient enrollment in clinical trials saw a significant decline, with a 74% decrease in new patient enrollments globally during the early months of the pandemic [5]. These statistics underscore the extensive impact of the pandemic on clinical research activities, highlighting the necessity for adaptive strategies to sustain research continuity during global health crises.

Researchers were unable to conduct experiments, assessments, or recruit participants [6], causing a pause in data gathering and gaps in numerous research investigations. Many medical institutions across the country supported the stay-at-home directive, requiring most non-essential employees to work remotely starting in March 2020. While remote research is possible, many studies cannot continue for extended periods without direct contact with participants [7].

Due to COVID-19, many researchers were at risk of losing their jobs because research activity decreased, and their roles may not be required anymore. Additionally, 55% of researchers on open-ended/permanent contracts felt that their job is under threat to some or a significant extent [8].

While the global impact of COVID-19 on medical research has been increasingly documented, there is limited evidence on how the pandemic has specifically affected research activities within the United Arab Emirates. Most existing literature related to the UAE has focused on clinical outcomes and public health responses, leaving a significant gap in understanding the internal disruptions faced by research institutions. Key aspects such as operational challenges, funding shifts, changes in research productivity, and the well-being of researchers remain underexplored.

To address this gap, this study aims to comprehensively evaluate the impact of the COVID-19 pandemic on medical research activities among faculty members at the University of Sharjah, specifically examining disruptions to research operations, changes in productivity, and encountered challenges. Additionally, the study aims to provide recommendations to mitigate barriers and enhance institutional preparedness for future public health emergencies. We hypothesize that the COVID-19 pandemic has significantly disrupted medical research among the University of Sharjah faculty, resulting in decreased productivity, operational interruptions, and increased barriers to conducting research.

## Materials and methods

Study design and participants

This study utilized a cross-sectional design to evaluate the impact of the COVID-19 pandemic on medical research activities at a single point in time. The study was conducted between February and March 2022 at the University of Sharjah, United Arab Emirates, specifically targeting faculty members from the Colleges of Medicine and Health Sciences. Ethical approval was obtained from the University of Sharjah Research Ethics Committee prior to initiation (REC-22-02-13-01-S).

Participants were selected through a convenience sampling method. All 126 eligible faculty members were invited via institutional email to participate voluntarily. Inclusion criteria required participants to be between the ages of 26 and 61 and to have engaged in research activities during the COVID-19 pandemic. Faculty members who were not involved in research during this period were excluded. A total of 101 responses were included in the final analysis. The sample size was determined using Cochran’s formula with a presumed prevalence of 50% to ensure maximum variability, and was adjusted for the finite population size of faculty members to enhance representativeness and statistical reliability.

Setting

The study was conducted at the University of Sharjah’s Colleges of Medicine and Health Sciences. The survey was disseminated electronically and remained accessible over a two-month period to allow sufficient time for participant engagement. The study period coincided with a transitional phase following the peak of the COVID-19 pandemic, which provided an appropriate context for retrospective reflection on its impact.

Variables

The primary outcome of interest was the perceived impact of the COVID-19 pandemic on the conduct of medical research. Key variables included demographic characteristics (e.g., age, sex, academic rank, and department), research-related metrics (e.g., number of ongoing projects, ability to access research facilities, and changes in publication timelines), and specific disruptions caused by the pandemic. All variables were pre-defined and categorized for statistical analysis. No diagnostic criteria were applicable due to the non-clinical nature of the study.

Data sources and measurement

Data were collected using a structured, self-administered online questionnaire developed by the research team following a comprehensive review of relevant literature (Appendices: Table 4). The questionnaire consisted of 26 items across three sections: demographics, research activity, and the pandemic’s effects on research conduct. To ensure content clarity and improve internal consistency, the tool was pilot tested among ten faculty members. While face validity was confirmed through expert feedback and pilot testing, no formal psychometric validation (e.g., Cronbach’s alpha, factor analysis) was conducted. Responses were collected using Microsoft Forms, which allowed for efficient data capture and storage.

Bias

Several measures were taken to reduce potential sources of bias. The inclusion of faculty from multiple health-related disciplines aimed to improve generalizability across the academic healthcare field. The survey’s anonymous nature was emphasized to reduce social desirability bias. Nevertheless, potential limitations include recall bias, given that participants were asked to retrospectively assess the pandemic's impact, and selection bias due to the reliance on a convenience sampling strategy.

Study size

Sample size estimation was based on Cochran’s formula, using a 50% expected prevalence to maximize sample variability. Given the finite faculty population of 126 individuals, a correction factor was applied, yielding a required minimum sample size of 101 respondents. This adjustment ensured that the study maintained adequate power for descriptive and inferential analysis.

Quantitative variables

Quantitative variables, such as age and number of research projects, were treated as continuous data and summarized using mean and standard deviation. Categorical variables, such as gender and academic rank, were summarized using frequencies and percentages. These groupings allowed for meaningful comparisons across relevant subgroups and facilitated bivariate analysis.

Statistical methods

All data were exported from Microsoft Forms to IBM SPSS Statistics version 22.0 for statistical analysis. Descriptive statistics were used to summarize participant characteristics and key variables. Univariate analyses included measures of central tendency (mean, median) and dispersion (standard deviation) for continuous data, and frequency distributions for categorical data. Bivariate analyses were conducted using Chi-square tests for categorical variables. The threshold for statistical significance was set at p < 0.05. Missing data were addressed through pairwise deletion, where analyses were performed using available responses per variable. No data imputation techniques were employed. Due to limitations in sample size, subgroup analyses and sensitivity analyses were not conducted.

## Results

Demographic data

A total of 101 faculty members participated in the study. The demographic breakdown included 61.4% males, and the predominant age group was 42-50 years (44.6%). Most participants were established researchers (40.4%) and predominantly from the College of Medicine (39.6%). Nearly half of the respondents (45.5%) were engaged in lab work, while 36.6% conducted community-based research, and 17.8% were involved in clinical trials. The majority (37%) reported using cross-sectional study designs, followed by case-control (19%), cohort (17%), and clinical trials (14%) (Table 1).

**Table 1:** Socio-demographic characteristics

Variables	n (%)
Gender	
Male	62 (61.39%)
Female	39 (38.61%)
Age Groups (Years)	
<41	28 (27.7%)
42-50	45 (44.6%)
>50	28 (27.7%)
Academic Position	
Professor	19 (18.81%)
Associate Professor	27 (26.73%)
Assistant Professor	29 (28.71%)
Lecturer	26 (25.74%)
Disciplinary Group	
College of Medicine	40 (39.6%)
College of Dentistry	22 (21.8%)
College of Pharmacy	10 (9.9%)
College of Health Sciences	29 (28.7%)
Level of Researcher	
Senior researcher	33 (33.3%)
Established researcher	40 (40.4%)
Early career researcher	26 (26.3%)
Type of Research	
Community-based research	37 (36.6%)
Lab work	46 (45.5%)
Clinical trials	18 (17.8%)
Study Design	
Cross-sectional	68 (37%)
Cohort	32 (17%)
Case-control study	35 (19%)
Clinical trials	25 (14%)
Other	24 (13%)

Research activities

Participants reported various impacts of COVID-19 on research-related activities. When asked about the effect of the pandemic on meetings and conferences, 44.6% indicated it was somewhat affected, while 36.6% were highly affected. Only 17.8% reported no impact.

In terms of funding opportunities, 40.6% of participants stated that funding was somewhat reduced, and 14.9% experienced a high reduction. A third (32.7%) reported no funding impact. Regarding access to facilities and medical centers, 29.7% reported high difficulty, and 42.6% experienced moderate difficulty, while 19.8% faced no issues. This disruption contributed to publication delays, with 12.9% highly affected and 42.6% somewhat affected. About 37.6% were unaffected.

When examining delays in fieldwork, 50.5% reported being somewhat affected and 31.7% highly affected. On research project counts, 26% reported a decrease, 50% a moderate change, and 24% no change. However, the number of research projects was not significantly associated with gender (p = 0.31, OR = 0.53, 95% CI: 0.21-1.31), college, researcher level, or type of research. Additionally, study design appeared to influence perceived research continuity. Compared to cross-sectional designs, cohort studies were associated with 76% lower odds of stable research output (OR = 0.24, 95% CI: 0.13-0.42, p = 0.557), case-control studies with 73% lower odds (OR = 0.27, 95% CI: 0.15-0.48, p = 0.693), and clinical trials with the lowest odds of continued research activity (OR = 0.17, 95% CI: 0.09-0.31, p = 0.483). Although none of these findings reached statistical significance, they suggest a consistent trend that study design type influenced research continuity.

Participants also identified the most disrupted research stages due to the pandemic. Data collection and subject recruitment were reported as the most affected, which aligns with pandemic-imposed restrictions. Additional challenges were noted in research collaboration, topic selection, and funding acquisition, reflecting broader disruptions across research phases.

**Figure 1:**
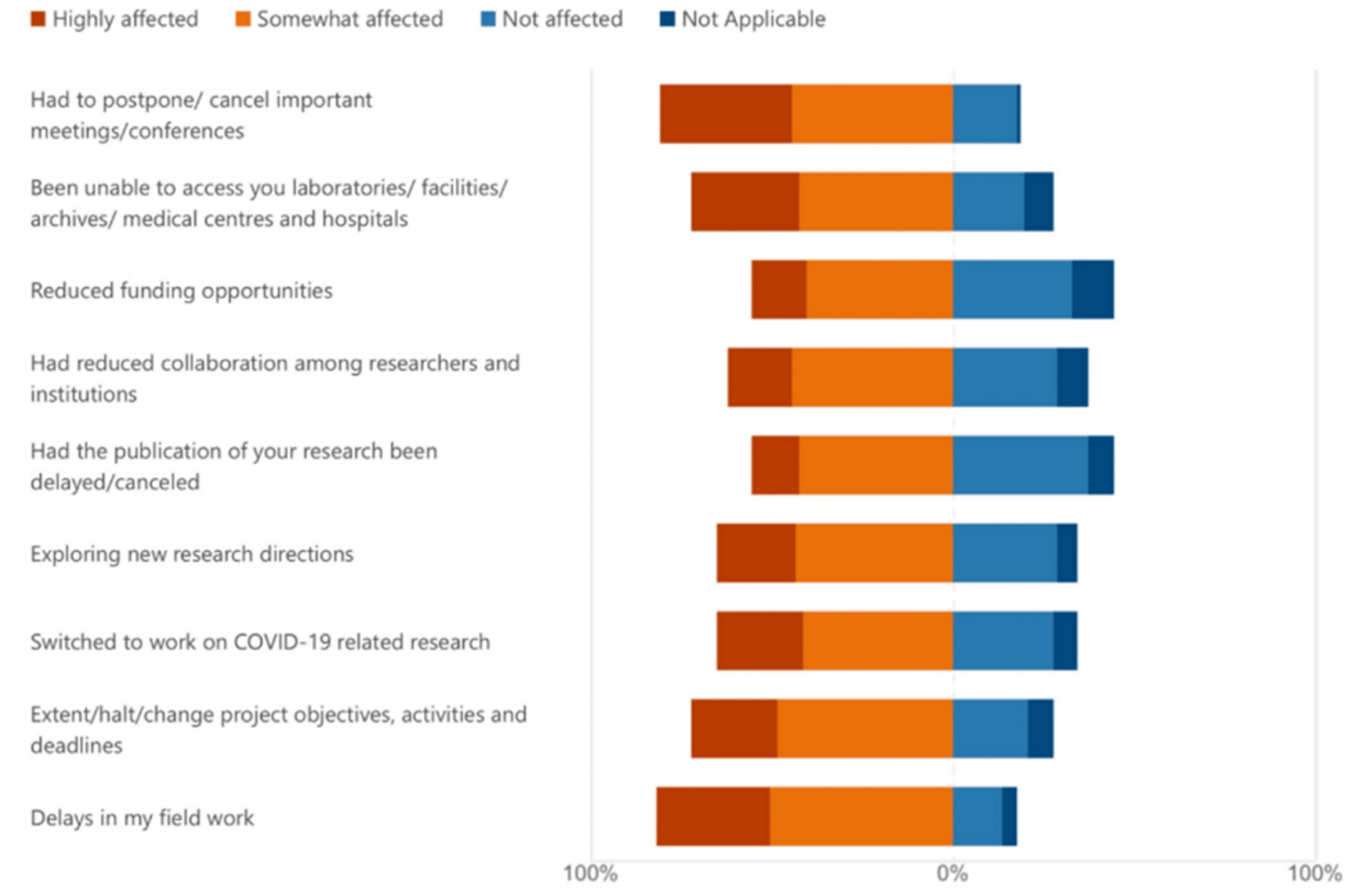
A graph representing the extent of change in research activities among researchers.

The change in research activities is presented in Figure 1. 

Effect on researchers

In evaluating how much time researchers allocated to their work during the pandemic, 39 participants reported spending 20% of their time on research, 32 allocated 50%, and 23 allocated 70%. A small number (6 participants) reported no time spent, and one dedicated 100%. There was no statistically significant association between time spent on research and researcher level or research type (Table 2). For example, senior researchers had an OR of 0.86 (95% CI: 0.31-2.42, p = 0.982) compared to early career researchers. Similarly, those engaged in lab work had an OR of 1.31 (95% CI: 0.55-3.13, p = 0.695) for spending more time on research than others (Table 2). 

**Table 2:** Bivariate analysis of the change in the time spent on research before and during the COVID-19 pandemic and its association with the level of researcher and type of research done by the researcher.

Variable	Time Spent on Research. Shown as n (%)		Chi-Square Test Value (χ^2^)	OR (95% C.I.)	p-value
	Decrease	No change			
Level of Researcher					
Early Career Researcher	12 (25%)	14 (27.5%)	-	-	-
Senior Researcher	14 (29.2%)	19 (37.3%)	0.0005	0.86 (0.31, 2.42)	0.982
Established Researcher	22 (45.8%)	18 (35.3%)	0.203	1.43 (0.53, 3.84)	0.652
Type of Research					
Community-based research	16 (33.3%)	21 (39.6%)	-	-	-
Lab work	23 (47.9%)	23 (43.4%)	0.154	1.31 (0.55, 3.13)	0.695
Clinical trails	9 (18.8%)	9 (17%)	0.034	1.31 (0.42, 4.06)	0.854

Regarding working hours, 28% reported a slight increase (0-10 hours per week), and 25% reported a substantial increase (>10 hours/week). Meanwhile, 25% reported no change, and 17% reported a decrease.

While 74.3% did not feel threatened about losing their research, a subset expressed concern about reduced work capacity due to home responsibilities. Specifically, 41.6% answered “somewhat”, 30.7% “no”, and 27.7% “yes”, suggesting moderate interference with work from home.

Another question asked if the subjects had a decrease in their work capacity due to other responsibilities, such as the house or children. 41.58% answered “Somewhat”, 30.69% answered “No”, and 27.72% answered “Yes,” which shows that there might be a slight decrease in the work capacity, which shows that staying at home, due to the COVID-19 pandemic, might not be the best option for working researchers.

The changes in the number of research activities conducted before and during COVID-19, stratified by demographic and professional variables, are presented in Table 3. Gender differences showed no significant association with research changes (p=0.31). Similarly, colleges (Pharmacy, Medicine, Dentistry, Health Sciences) and researcher levels (Early Career, Senior, Established) did not exhibit significant variations (p>0.05 for all). Study designs, including cross-sectional, cohort, case-control, and clinical trials, also showed no statistically significant differences in research activity changes (p>0.05 for all). Overall, the data suggest that the pandemic's impact on research productivity was consistent across these variables.

**Table 3:** Bivariate analysis of the change in the number of research studies conducted before and during the COVID-19 pandemic and its association with demographic variables.

Variable		Has the number of your research conducted before and during COVID-19 changed? Shown as n (%)			Chi-square test value (χ^2^)	OR (95% C.I.)	p-value
		Yes	Somewhat	No			
Gender							
Male		13 (50%)	32 (62.7%)	17 (70.8%)	2.37	0.53 (0.21, 1.31)	0.31
Female		13 (50%)	19 (37.3%)	7 (29.2%)			
College							
Pharmacy		3 (30%)	4 (40%)	3 (41.7%)	-	-	-
Medicine		10 (25%)	20 (50%)	10 (25%)	0.32	0.78 (0.17, 3.59)	0.852
Dentistry		6 (27.3%)	11 (50%)	5 (22.7%)	0.31	0.88 (0.17, 4.54)	0.856
Health Sciences		7 (24.1%)	16 (55.2%)	6 (20.7%)	0.71	0.74 (0.15, 3.67)	0.700
Level of Researcher							
Early Career Researcher		7 (26.9%)	14 (53.8%)	5 (19.2%)	-	-	-
Senior Researcher		9 (27.3%)	17 (51.5%)	7 (21.2%)	0.04	1.02 (0.32, 3.24)	0.978
Established Researcher		10 (25%)	18 (45%)	12 (30%)	0.99	0.90 (0.29, 2.78)	0.611
Study Design							
Cross-sectional	Yes	20 (76.9%)	34 (66.7%)	14 (58.3%)	1.981	2.38 (0.70, 8.07)	0.371
	No	6 (23.1%)	17 (33.3%)	10 (41.7%)			
Cohort	Yes	8 (30.8%)	15 (29.4%)	10 (41.7%)	1.172	1.61 (0.50, 5.14)	0.557
	No	18 (69.2%)	36 (70.6%)	14 (58.3%)			
Case-Control	Yes	9 (34.6%)	20 (39.2%)	7 (29.2%)	0.735	0.78 (0.24, 2.57)	0.693
	No	17 (65.4%)	31 (60.8%)	17 (70.8%)			
Clinical Trials	Yes	8 (30.8%)	14 (27.5%)	4 (16.7%)	1.456	2.5 (0.50, 12.50)	0.483
	No	18 (69.2%)	37 (72.5%)	20 (83.3%)			

## Discussion

The global COVID-19 pandemic has broadly impacted research activities worldwide. This study comprehensively evaluated the impact of the COVID-19 pandemic on medical research activities among faculty members at the University of Sharjah, specifically assessing disruptions to research operations, changes in productivity, and the challenges encountered. The findings showed a significant interruption of research during the pandemic, highlighting several challenges and issues faced by researchers that limited the conduct of research projects, as has been reported globally. Major challenges encountered included the postponement of meetings and conferences, lack of access to facilities, delays in data collection and publication, participant recruitment, and a decrease in funding [8-11].

Researcher’s demographics

Our study, senior researchers reported greater difficulty in accessing facilities, archives, and medical centers during the pandemic, which had a significant impact on their research activities. This may be attributed to senior researchers’ reliance on physical facilities for advanced research activities. This is consistent with another study, which showed a higher impact on researchers with advanced qualifications [12].

Gender inequality was not an issue in our study. Both male and female researchers reported similar levels of disruption to their research activities, in contrast to other studies that showed that females had a harder time conducting their research projects during the pandemic due to childcare or schoolwork responsibilities [13].

Research productivity

The global scientific community faced many challenges due to the pandemic, which had a profound impact on the continuity and productivity of research, leading to widespread delays in research activities, particularly in clinical trials and lab work. In our study, 44.5% were somewhat affected by the postponement of meetings, conferences, and collaborations, with 36.6% being highly affected. Such delays were also reported by other researchers. A study conducted in Latin America documented disruptions in the conduct of clinical trials due to the lack of collaboration and coordination at national and regional levels. This hindered the capability of suggesting evidence based on research results [14].

One of the most affected areas was access to research facilities and archives. The temporary closure of laboratories, libraries, and medical centers impacted data collection and publication timelines, mirroring global trends. Prior studies from different parts of the world described this issue as a main problem, as they experienced delays in data collection due to lockdown restrictions, particularly in fields requiring direct contact with participants and laboratory access [13-16]. This has significantly affected clinical science due to many clinical trials being paused and the difficulty in reaching patients. The FDA provided guidance and recommendations to sponsors to address common challenges like quarantine and travel limitations to ensure the safety of participants and the integrity of clinical trials [17]. Those who reported no impact on access to archives and medical centers possibly had desk-based or computational work.

Funding

A critical issue faced by researchers was the reduction in funding opportunities during the pandemic. This was also reported by institutions worldwide, as they faced financial shortages due to the reallocation of research budgets toward COVID-19 projects [13]. This led to a negative impact on researchers involved in non-COVID-19 research. The 32.67% of respondents who reported no effect on funding opportunities in our study may have benefited from institutional support or may have already shifted to COVID-19-related research, as reported by 27.7% of respondents. This shift was expected, given the urgent need for new clinical science and knowledge to provide interventions and control the pandemic. Global research output reflected this trend, with the number of non-COVID-19-related research papers decreasing by 18% from February 1, 2020, to December 31, 2020 [10].

Our study showed diverse experiences among the academic community during the pandemic. While some researchers faced significant barriers that prevented them from conducting their research, others were able to adapt and continue their projects, as seen with 27.7% of our participants.

Effect on researchers’ working time

The impact on the researchers’ work habits varied significantly. Thirty-nine respondents allocated 20% of their time to research, while 23 respondents dedicated up to 70% of their time. These disparities reflect possible differences in the nature of research, responsibilities at home, and personal circumstances. The lack of access to facilities and the inability to conduct the research remotely may have contributed to the decrease in working hours. A study suggested that researchers with caregiving responsibilities (i.e., women with young children at home) reported a challenge during lockdowns, finding it difficult to maintain productivity levels while managing household responsibilities, which reduced time dedicated to research. Alternatively, others indicated an increase in working hours, possibly due to the additional tasks of rescheduling research activities and seeking alternative methods for research conductance or funding resources.

Interestingly, the majority of our participants (74.26%) did not feel that their research progress was threatened. In contrast, other studies showed that research trainees and established career researchers feared for the future of their careers and losing their jobs, especially if they did not prioritize COVID-19-related topics [18].

Affected stage of research

In terms of research stages, data collection and subject enrollment were most affected during the pandemic, which is expected given the social distancing regulations and lockdowns. A study similarly noted a delay in clinical trials due to the inability to recruit participants [19].

Limitations

The study design was cross-sectional, capturing the impact of COVID-19 on research activities at a single point in time. This limits the ability to assess changes over time or understand ongoing effects. A longitudinal approach would have provided deeper insight into evolving research challenges. Furthermore, although convenience sampling allowed efficient data collection during pandemic restrictions, it introduces potential selection bias. While our sample proportionally represented faculty from medicine (39.6%), dentistry (21.8%), and health sciences (28.7%) - mirroring the institutional distribution - participants may have been skewed toward those with internet access or a stronger motivation to participate, potentially those more affected by the pandemic.

Another limitation is the relatively small sample size, though it still offers valuable insights into the pandemic's impact on research. Additionally, including only University of Sharjah faculty members limits the generalizability of findings to other institutions within the UAE or internationally.

Importantly, no qualitative data were collected or analyzed in this study. As such, we were unable to explore in depth why certain researchers were more affected than others, which could have provided important contextual understanding. Future research could benefit from including open-text responses or interviews to capture this dimension.

Finally, no multivariate analysis was conducted to adjust for potential confounding factors such as age, gender, research type, or academic seniority. Including such analyses in future studies could offer a clearer picture of which factors most strongly influenced research disruption during the pandemic.

Despite these limitations, the study provides meaningful insights into the challenges faced by researchers and highlights areas to strengthen research continuity in times of crisis.

Future research recommendations

Several recommendations can be made to mitigate the barriers identified during the pandemic. Institutions should provide better access to digital resources like archives, data platforms, and collaboration tools to support remote research and reduce disruptions during emergencies. Furthermore, creating contingency plans for research activities and training researchers on how to adapt during global health emergencies would help prepare them for future crises. Additionally, funding agencies-including governments, institutions, and the private sector-must collaborate to create resilient funding mechanisms and adaptable models that allow researchers to easily pivot to urgent projects and ensure the continuity and growth of medical research.

Future research could examine the long-term effects of the pandemic on research productivity by conducting longitudinal studies among different levels of researchers. Stratified or random sampling methods could be used to reduce selection bias. Comparative studies between different countries may also offer a better understanding of disparities across the globe. Additionally, further studies are needed to investigate the effectiveness of remote research tools during crises.

## Conclusions

To the best of our knowledge, this is the first study conducted in the UAE that explores the impact of COVID-19 on medical research activities at an institutional level. Focusing on researchers at the University of Sharjah, we captured a diverse range of perspectives across different researcher levels, disciplines, and study designs. Our findings highlight key challenges experienced during the pandemic, including delays in data collection, limited collaboration, restricted access to research facilities, and funding shortages. While the scope of the study is limited to a single institution, the insights gained may inform local policy decisions aimed at strengthening institutional resilience and research continuity during future public health emergencies. Broader, multi-institutional studies are needed to better understand the nationwide impact.

## Appendices

**Table 4:** Questionnaire

Section	Question	Response Option 1	Response Option 2	Response Option 3	Response Option 4	Response Option 5	Response Option 6
Consent	I have read and understood the information above and agree to take part in the study	Yes	No				
A. Demographics	Age/DOB	Open-ended					
	Gender	Male	Female				
	Current Position	Professor	Associate professor	Assistant professor	Lecturer		
	Level of Researcher	Senior researcher	Established researcher	Early career researcher	Others, specify: _______		
	Research Disciplinary Group	College of medicine	College of dentistry	College of health sciences	College of pharmacy		
	Type of Research	Community-based research	Lab work	Clinical trials			
	Study Design (Multiple Answers Allowed)	Cross-sectional	Cohort	Case-control study	Clinical trials	Others, specify: _______	
B. Impact of COVID-19 on Research Activities	Postponed/canceled important meetings/conferences	Highly affected	Somewhat affected	Not affected	Not applicable		
	Unable to access laboratories, archives, medical centers	Highly affected	Somewhat affected	Not affected	Not applicable		
	Reduced funding opportunities	Highly affected	Somewhat affected	Not affected	Not applicable		
	Reduced collaboration among researchers and institutions	Highly affected	Somewhat affected	Not affected	Not applicable		
	Delayed/canceled research publication	Highly affected	Somewhat affected	Not affected	Not applicable		
	Exploring new research directions	Highly affected	Somewhat affected	Not affected	Not applicable		
	Switched to COVID-19 related research	Highly affected	Somewhat affected	Not affected	Not applicable		
	Change in project objectives, activities, and deadlines	Highly affected	Somewhat affected	Not affected	Not applicable		
	Delays in fieldwork	Highly affected	Somewhat affected	Not affected	Not applicable		
	Change in number of researches conducted before and during COVID-19	Yes	Somewhat	No			
	Impact of distancing guidelines on recruiting participants (Scale 0-10)	0 (Not affected)	1-9	10 (Very affected)	Not applicable		
	Impact on follow-up of recruited participants (Scale 0-10)	0 (Not affected)	1-9	10 (Very affected)	Not applicable		
	Percentage of time allocated to research during COVID-19	100%	70%	50%	20%	0%	
C. Impact on Researchers	Change in weekly working hours since lockdown	Large decrease (>10 hours)	Decrease (0-10 hours)	No change	Increase (0-10 hours)	Large increase (>10 hours)	
	Average weekly working hours	0-10 hours	11-20 hours	21-30 hours	31-40 hours	>40 hours	
	Concern about research area being lost	Yes	No				
	Level of worry regarding research progression (Scale 0-10)	0 (Not worried)	1-9	10 (Extremely worried)			
	Decrease in work capacity due to other responsibilities	Yes	Somewhat	No			
	Impact of working from home on research activities	Positively	Negatively	No impact			
	Research stages significantly affected due to COVID-19 (Multiple answers allowed)	Choosing the topic	Selecting research design	Data collection tool and method	Recruiting subjects	Collaboration with other researchers or faculties	Funding
